
JOURNAL INFORMATION
==============================
NLM Title Abbreviation: Buffalo Med J Mon Rev Med Surg Sci
NLM Journal ID: 0004276

The Buffalo Medical Journal and Monthly Review of Medical and Surgical Science

Publisher: Biomedical Journal Digitization Project

ARTICLE INFORMATION
==============================
PMCID: PMC8932457
PMID: 35376954
Article version: 1
Subjects: Editorial Department

The Physician's Diary for 1859

Print publication date: 1858 Dec
Volume: 14
Issue: 7
First page: 448
Last page: 448
License: This work is free of known copyright restrictions. For more information, please see PMC Back Issue Digitization.
License URL: https://creativecommons.org/publicdomain/mark/1.0/

==============================

*The Physician's Diary for* 1859. Buffalo: Phinney & Co., publishers, No. 188 Main street. 1859.—We have just received this admirable Physician's Diary for 1859, and hasten to notice its appearance. This has been the companion of most if not all our physicians for the past year or more, and we, for one, can testify to its usefulness and convenience. It is of a size sufficiently small to be carried in the pocket without inconvenience, and yet is so admirably arranged as to contain all the notes which we wish to make in such a form, It is so well known to our-readers that it is almost unnecessary to recite what it contains. Every physician should commence the year with one. They may be obtained of different sizes, from Phinney & Co., of this city. The contents are, An Almanac, List of Poisons and their Antidotes, Visiting List and Medical Record, Obstetrical Record, Nurses' Addresses, List of Things Lent, and Bills to be Presented.

---

*To Exchanges and Sucscribers.*—We are very short of the August number for 1858, of our Journal, it having been nearly all sent off by mistake, and should be glad to credit fifty cents on the subscription of any person who does not bind the Journal and will mail this No. to us. $2 and the August number for 1858, will be received for subscription for the next volume. If any of our exchanges can spare us this number, of which every one counts with us, and will send it to us, it will do us a great service.

---

*Congenital Fissure of the Sternum.*—We have received a pamphlet containing extracts from the Album of E. A. Groux, who has a congenital fissure of the sternum, and suppose that the profession of this city will soon have an opportunity of examining this remarkable case. The extracts are interesting observations on his case by some of the most distinguished German, Spanish, French and English physicians, and indicate the great interest which Mr. Groux has excited among members of the profession wherever he has exhibited himself.

---

☞ Prof. Flint left Buffalo in the early part of November to enter on the duties of his Chair in the New Orleans School of Medicine. He expects to return in the latter part of February next. His correspondents, in the meantime, will please direct their letters to him at New Orleans.
